# The Application of Regional Anaesthesia in Türkiye: National Survey Study

**DOI:** 10.4274/TJAR.2025.251900

**Published:** 2025-10-14

**Authors:** Elvin Kanat, Zeynep Çağıran, Nezih Sertöz

**Affiliations:** 1 Ege University Faculty of Medicine Department of Anaesthesiology and Reanimation, İzmir, Türkiye

**Keywords:** Adjuvant drugs, local anaesthetics, multimodal analgesia, regional anaesthesia

## Abstract

**Objective:**

This study was designed to determine why anaesthesiologists working in various institutions in our country prefer current regional anaesthesia methods and to evaluate the use and prevalence of ultrasonography in these methods.

**Methods:**

A questionnaire created on SurveyMonkey.com was sent electronically or face-to-face to anaesthesiology and reanimation physicians working in different provinces of our country, and they were asked to fill it out. The survey was intended to be administered to at least 200 volunteer anaesthesiologists. The questionnaire consisted of 34 questions, including demographic characteristics, neuraxial block and peripheral nerve block (PNB) applications, drug choices, preferences in paediatric cases, training, and safety measures.

**Results:**

A total of 215 anaesthesiologists participated in our questionnaire. 39.2% were working in a university hospital, and 38.2% were working in a training and research hospital. PNB training was received by 89.2% of the participants during specialty training. For analgesic purposes, the interscalene block was preferred for shoulder surgery (57.4%), the axillary block for elbow, forearm, and hand surgery (49.8%), the erector spinae plane block for thoracic surgery (33.8%), and the transverse abdominis and rectus block for open abdominal surgery (51.5%).

**Conclusion:**

Regional anaesthesia is an essential part of multimodal analgesia and is used both as an anaesthetic and analgesic in routine practice. In recent years, many new techniques have been utilized as a result of advancements. However, for these to be implemented in practice, up-to-date information should be closely followed, and anaesthetists should be supported in terms of training and equipment.

**Main Points**

• Regional anaesthesia (RA), particularly peripheral nerve blocks, is increasingly used and becoming more popular among young physicians. Its importance in reducing opioid consumption, enhancing multimodal analgesia, and shortening hospital stays has been recognized.

• Despite its benefits, RA is sometimes limited by equipment shortages and insufficient training. These barriers have been partly overcome, but hospitals providing training still need additional resources, particularly ultrasound equipment, to better support RA education and practice.

• RA plays a significant role in postoperative pain management, especially in reducing opioid consumption, which can lead to fewer side effects and complications. Additionally, RA contributes to reduced healthcare costs through its efficiency in pain management and shorter recovery times.

• The safety of RA procedures is paramount. One of the recommended safety measures is having intralipid solutions available in clinics performing these procedures to mitigate the risks associated with local anaesthetic toxicity.

• This study aims to provide a reference for future research, offering insights into the use of RA applications. This can help further develop protocols, improve training, and ensure patient safety.

## Introduction

Regional anaesthesia (RA) techniques are commonly used both for anaesthesia during surgical interventions and for postoperative pain management. The advancements in needles and catheters used in RA, as well as the introduction of new and safer local anaesthetics into clinical practice, have increased interest in RA.^1^ Additionally, in recent applications of ultrasound-(USG) guided RA, target structures, anatomical relationships, and drug distribution can be visualized.^2^ Therefore, it has significantly expanded the scope of anaesthetists’ practices, leading to the introduction of new blocks in clinical settings and changes in preferences between existing blocks.^3^ In our country, basic and advanced clinical USG courses for anaesthesiologists are organized by anaesthesia associations to support USG and RA education. However, there is no comprehensive recent study in our country investigating RA applications, USG usage, or the changes caused by USG in clinical practice.

This study aims to explore the preferences of anaesthesiologists regarding RA techniques and the extent to which USG is utilized in these practices. Specifically, it seeks to identify factors influencing these preferences and assess how widespread the use of USG is in the administration of RA across different institutions.

## Methods

The survey study titled “Regional Anaesthesia Applications in Türkiye: A National Survey Study” was conducted between September 2023 and March 2024, after obtaining approval from the Ege University Medical Research Ethics Committee (approval no.: 2023-0291, dated: 11.05.2023). The survey, created on SurveyMonkey.com, was distributed electronically or in-person to anaesthesiology and reanimation specialists working in various provinces of Türkiye. The survey was designed to be filled out by a minimum of 200 anaesthesiologists. The purpose and objectives of the study were explained to the participating anaesthesiologists. Participation in the study was entirely voluntary. The survey consists of 34 questions, addressing topics such as demographic characteristics, neuraxial block and peripheral nerve block (PNB) applications, drug selection, education, and safety measures (Appendix 1: Survey form).

The first six questions were aimed at identifying the demographic characteristics of the participants, (age, gender, institution where specialist training was received, current institution, job title, and education related to peripheral block application). Questions 7-14 asked about RA preferences: in upper extremities, lower extremities, abdominal surgery, and day-case surgeries. Questions 15-24 addressed the choice of needles, application methods, and safety precautions taken in neuraxial anaesthesia. Questions 25-29 inquired about the drugs used for peripheral blocks, methods, precautions taken to avoid nerve damage, and RA complications. Questions 30-31 asked about sedation preferences during RA. Finally, questions 33 and 34 evaluated anaesthesiologists’ RA preferences for paediatric patients.

### Statistical Analysis

Our analyses were performed using SPSS 26.0 software, with a 95% confidence level. In the analyses, frequency and percentage values were calculated for categorical variables, while the mean and standard deviations were computed for age. The relationship between job title, current institution, and categorical variables was analyzed using the chi-square test.

## Results

A total of 215 anaesthesiologists started the survey, with 204 completing all questions. 32.4% of participants were 30 years old or younger, 37.3% were between 31 and 40 years old, and 30.4% were 41 years old or older. The average age was 35.98, with a standard deviation of 7.85 (Table 1).

The most common reason for not choosing RA is the patient’s refusal, which was cited by 85.8% of respondents, followed by insufficient time (37.7%) and concerns about complications (22.1%).

Among the most common positions for performing routine neuraxial techniques, sitting (53.9%) and condition-dependent positions (36.8%) are prominent. For spinal anaesthesia, sharp-tipped needles (73.0%) and 25G needles (68.1%) are generally preferred. The most commonly used local anaesthetic for spinal anaesthesia is bupivacaine, which accounts for 99.0% of cases.

The most frequently used method for defining the epidural space is the loss of resistance to fluid (89.2%), while the most commonly used drugs for epidural test doses are 3 mL of 2% lidocaine (52.0%) and 3 mL of 1.5% lidocaine with 15 µg of adrenaline (41.2%). The most commonly used drugs for postoperative epidural analgesia are opioids and local anaesthetics, used together (73.5%) (Table 2).

The percentage of people using epidural catheters for postoperative analgesia is 88.7%, while 39.2% use adjunct medications for spinal block. Among the adjunct medications used, fentanyl (70.5%) and morphine (29.5%) are the most common (Table 3).

There is a request to rank the frequency of complications after RA. The most common complication is postspinal headache, at 67.2%. The second most frequent is Horner’s syndrome, at 32% (Table 4).

When examining the postoperative RA method preferences in paediatric patients, peripheral block (40.7%) and caudal block (39.7%) are the most commonly preferred methods. These are followed by the epidural catheter procedure (3.4%) and spinal anaesthesia (2.5%), demonstrating their respective proportions. Additionally, the proportion of those who do not prefer regional techniques in children is also noteworthy, with 33.3% (Table 4).

When examining the age limits for applying regional analgesia in paediatric patients, it is observed that a significant portion of participants (45.6%) do not apply it to very young children. Some participants (22.1%) stated that they applied regional analgesia to all children without specifying age limits, while others (32.4%) mentioned that they did not apply regional analgesia to paediatric patients (Table 4).

There is a significant relationship between the institution where the participant works and the percentage of surgeries performed under RA (*P *< 0.05). According to this, 43.8% of those working in university hospitals perform surgeries under RA in 40-60% of cases; 59.0% of those working in teaching and research hospitals perform them in more than 60% of cases; 44.7% of those working in state hospitals perform them in 40-60% of cases; and 62.5% of those working in private hospitals perform them in 40-60% of cases.

There is a significant relationship between the institution where the participant works and the preference for PNB or catheter infusion for postoperative analgesia (*P *< 0.05). According to this, 53.8% of those working in university hospitals, 76.9% of those in teaching and research hospitals, 60.5% of those in state hospitals, and 87.5% of those in private hospitals prefer PNBs or catheter infusions for postoperative analgesia (Table 5).

There is a significant relationship between the institution where the participant works and the first choice of analgesia in open abdominal surgery (*P *< 0.05). According to this, 63.8% of university hospital workers and 55.3% of state hospital workers prefer transversus abdominis and rectus blocks, while 55.1% of teaching and research hospital workers and 62.5% of private hospital workers prefer epidural analgesia.

There is a significant relationship between the institution where the participant works and the most commonly used technique in PNB (*P *< 0.05). According to this, 78.8% of those working in university hospitals use USG plus nerve stimulator (USG+NS), 60.3% of those in teaching and research hospitals use USG, 42.1% of those in state hospitals use either USG+NS or USG, and 50.0% of those in private hospitals use USG.

## Discussion

RA has become a widely used technique in recent years, both worldwide and in Türkiye. The growing popularity of RA can be attributed to its advantages, including the reduction in opioid consumption, decreased stress response during surgery, reduced intraoperative and postoperative blood loss, provision of high-quality analgesia specific to the region, enhanced early mobilization and rehabilitation, and the ability to communicate with the patient and better guide treatment due to the patient being conscious. Developments in RA techniques, such as advancements in needles, catheters, and imaging methods, have further contributed to its increased popularity.

The younger population has followed the developments in RA more closely. It is believed that the higher percentage of residents who are still in ongoing training contributed to this trend. A similar age, professional experience, and role distribution was found in a thesis study conducted in Türkiye.^4^ When the distribution of participants by institution was compared, it was found to be similar to that in the study by Gürkan et al.^3^ and the distribution of aneasthesiologists in the country. The basis of RA education was based on specialist training. RA education is of a certain standard in all hospitals providing training and is supported by practical training by the Regional Anesthesia Association. The facilities of hospitals, patient profiles, and the knowledge, skills, interests, and experience of the educators may vary, which could make standardization more challenging.

In postoperative pain management guidelines, the importance of multimodal analgesia is emphasized and its use is recommended. The widespread and successful application of RA and analgesia methods has shown positive outcomes in reducing opioid use. Regarding the participants’ preferences for postoperative analgesia, the most common choice was acetaminophen and non-steroidal anti-inflammatory drugs, followed by PNBs or catheter infusion (Table 6).

 The widespread use of PNB is thought to be due to its anaesthetic properties and effectiveness in pain management.

Postoperative pain management following shoulder surgery can be challenging. A systematic review of 2,391 articles on postoperative pain following shoulder surgery suggested that interscalene blocks were ideal for early postoperative analgesia.^5^ In a study by Lin et al.^6^, the interscalene block was identified as the most beneficial PNB for shoulder surgery, while the supraclavicular block was suggested as an alternative. In our study, more than half of the participants preferred the interscalene block as their first choice. The axillary block, which is simple, easy to apply, and safe, is the most commonly used PNB, especially for elbow, forearm, and hand surgeries.^3, 7^

A meta-analysis published in 2024 highlighted the importance of the Enhanced Recovery After Surgery protocol, in improving recovery after hip and knee surgeries, with nerve blocks and infiltration analgesia, which are key components of this protocol.^8^ A significant portion of participants in our study preferred spinal and epidural anaesthesia for postoperative analgesia in both surgeries. In recent years, there has been a trend towards peripheral blocks due to undesirable complications of neuraxial anaesthesia.

Thoracic epidural analgesia has long been the gold standard for multimodal analgesia in thoracotomy.^9^ With the widespread use of USG, ESP block applications are used more frequently than the more invasive thoracic epidural and paravertebral applications. The ESP block has also been shown to improve chronic post-thoracotomy pain syndrome in patients, weeks after surgery.^10^ Regional blocks play a significant role in abdominal surgery, improving postoperative recovery.^11^ Recently, the interest in transversus abdominis plane blocks has increased substantially, and they have been shown to provide sufficient analgesia for abdominal surgery.^12^

In our study, the most common reason for not choosing RA was found to be the patient’s refusal. This result is consistent with studies conducted in Türkiye.^3, 4^ However, a study in China found that the most common reason for not using RA was concern about complications.^13^ In our study, concern about complications was found to be the third most common reason, at a rate of 22.1%. We think that real-time block application with USG in RA reduces the concern for complications.

The most common position for performing routine neuraxial techniques was found to be the sitting position. A study by Aksu et al.^14^ showed that the interspinous distance was wider in the sitting position than in the lateral decubitus position. This suggests that the sitting position may enhance the success of neuraxial blocks. It was observed that spinal anaesthesia was typically performed using sharp-tipped 25G needles, with bupivacaine being the most commonly preferred local anaesthetic due to its long duration of action and availability. A study conducted in India in 2021 yielded results similar to those in our country.^15^ In defining the epidural space, the loss of resistance using fluid, was the most common method, although this technique requires experience and is subjective. In inexperienced hands, the failure rate can be as high as 15%. Using air, on the other hand, is associated with adverse effects such as headache, nerve injury, and insufficient spread of the medication, which are not observed with fluid. To increase success rates, new techniques with high sensitivity and specificity should be preferred.^16^ Many participants indicated that they use a test dose, with 3 mL of 2% lidocaine being the most commonly used.

The use of epidural catheters for postoperative analgesia was found to be very common. Their widespread use is likely due to their ability to provide both anaesthesia and analgesia. The combination of opioids and local anaesthetics was the most common choice for this purpose. The use of adjuvants can enhance the effect of local anaesthetics and prolong intraoperative and postoperative analgesia. Studies have shown that a smaller dose of bupivacaine is associated with less hypotension and faster recovery.^17^ Fentanyl was the most commonly used adjuvant. However, prolonged opioid use can lead to adverse effects like respiratory depression, nausea, and vomiting.

In PNB, the use of blind techniques has largely been abandoned due to the serious complications they can cause and the widespread use of USG. In comparison with other countries, blind techniques were still used in China, NS was common in Greece, and USG was the preferred method in India.^1, 13, 18^ It was observed that the choice of local anaesthetic in PNB was bupivacaine-prilocaine or bupivacaine and lidocaine combination, which provides a rapid onset of action and long duration of effect. It is advisable that lidocaine should be preferred because of the methemoglobinemia-inducing effect of prilocaine. Fentanyl and dexamethasone were commonly used as adjuvants in PNBs, helping to reduce the required dose of local anaesthetics while maintaining effective anaesthesia without enhancing motor blockade. Despite the benefits of these drugs, many are not approved by the Food and Drug Administration, and caution should be exercised in their use.^19^

The incidence of local anaesthetic systemic toxicity (LAST) in PNBs was found to be 20/10,000, while in epidural blocks, it was 4/10,000.^20^ LAST is a serious and life-threatening complication that requires immediate intervention. The standard treatment for LAST is the administration of Intralipid solution, which should be readily available in clinics performing RA procedures, with expiration dates regularly monitored. Our study found that most clinics had intralipid solutions available.

It was noted that sedation was frequently used both before and after RA procedures. Sedation helps ensure patient cooperation, prevent sudden movements, and enhance procedural safety. However, some participants avoided sedation, arguing that it might mask the early warning signs of LAST.^21^

In our study, the use of peripheral block catheters for postoperative analgesia was found to be rare, and in most clinics, they were not used. (Table 7). We believe that the low usage could be attributed to a lack of materials and insufficient knowledge and training about their safety. Similar findings have been reported in other national studies.^1, 13^

In our study, caudal blocks were the most commonly used peripheral blocks in children, but a significant proportion of participants also reported not preferring RA in paediatric cases. Most participants indicated that age limits were crucial in paediatric RA applications, with many avoiding the use of paediatric RA applications in very young children. The difficulty in recognizing surface landmarks, as well as the variability in the depth and location of nerves in growing children, makes RA more challenging. However, these difficulties have been overcome with the use of USG.^22^

RA application rates were higher in educational and research hospitals and university hospitals, likely due to the training provided to residents and the diversity of cases encountered. In contrast, PNB and catheter infusions were more common in private hospitals, likely due to better availability of equipment. However, the small number of participants from private hospitals in this study may not fully reflect the actual distribution.

### Study Limitations

There are several limitations to our study. We created a heterogeneous group of experts in RA, experienced physicians outside of RA, and resident physicians in training. The survey was administered both online and face-to-face and using a single method, particularly face-to-face administration, could have reduced measurement errors. Due to the low number of participants, we believe the results may not fully reflect national preferences, and larger studies with more participants are needed.

## Conclusion

RA is a widely used and developing field in our country frequently preferred by young physicians. The importance of supporting RA education during residency training has become more prominent. It was observed that the limited use of PNBs due to equipment shortages and insufficient training, has been partially overcome, and their application has become more widespread. Hospitals providing training should be supported with the necessary equipment and USG resources.

RA plays a crucial role in postoperative multimodal analgesia. By reducing opioid consumption and providing long-lasting analgesia, RA contributes to shorter hospital stays and reduced healthcare costs. The most common contraindication for RA was found to be the patient’s refusal. The application rate can be increased by adequately explaining the procedure to the patient and discussing its advantages. It is essential to implement the recommended safety measures for RA, and clinics where these procedures are performed must have intralipid solutions available. This would likely reduce morbidity and mortality associated with local anaesthetic toxicity.

Our study can serve as a reference for future research, providing valuable insights into the detailed use of RA applications.

## Ethics

**Ethics Committee Approval:** Ethical approval was obtained from the Ege University Medical Research Ethics Committee (approval no.: 2023-0291, dated: 11.05.2023).

**Informed Consent:** Survey study.

## Supplementary Materials

Appendix 1 Survey Questions of Our Study
https://d2v96fxpocvxx.cloudfront.net/a2440bda-5c5c-4e7b-8a75-abf1691c9260/content-images/2bea5f97-bf7e-4c08-afd2-2afa316f7d7b.pdf

## Figures and Tables

**Table 1. Demographic Characteristics of Participants:** 

**Category**	**n (%)**	**Category**	**n (%)**
**Age**	-	**Institution**	-
30 years or younger	66 (33)	University hospital	80 (39)
31-40 years	76 (37)	Training and research hospital	78 (38)
41 years or older	62 (30)	State hospital	38 (19)
-	-	Private hospital	8 (4)
**Gender**	-	**Years of professional experience**	
Male	85 (42)	Less than 5 years	75 (37)
Female	119 (58)	5-10 years	54 (26)
Position	-	10-15 years	24 (12)
Resident	85 (42)	More than 15 years	51 (25)
**Specialist**	**92 (45)**	**Peripheral block training (multiple choices allowed)**	
Doctor of medical sciences	8 (4)	Specialization training	182 (90)
Associate professor	11 (5)	Association courses	75 (37)
Professor	8 (4)	Fellowship programs	32 (16)
-		Internet	80 (39)
-		Other (e.g., textbooks, current literature)	4 (2)

**Table 2. Preferences in Neuraxial Block Technique and Postoperative Analgesia:** 

-	**n (%)**
**Reason for not choosing RA** **(multiple choices allowed)**	
Insufficient time	77 (37)
Patient refusal	175 (86)
Low success rate	9 (4)
Concerns about complications	45 (22)
**Most commonly used position for routine neuraxial technique**	
Sitting	110 (54)
Lateral decubitus	19 (9)
Prone	0 (0)
Dependent on the patient’s condition	75 (37)
Preferred needle type for spinal anaesthesia	
Pencil point (side-hole) needle	55 (27)
Sharp-pointed (Quincke) needle	149 (73)
**Preferred needle size for spinal anaesthesia** **(multiple choices allowed)**	
22G	55 (27)
25G	139 (68)
26G	133 (65)
27G	33 (16)
Most frequently used local anaesthetic for spinal anaesthesia (multiple choices allowed)	
Bupivacaine	202 (98)
Ropivacaine	2 (1)
Lidocaine	2 (1)
**Method used for identifying the epidural space** **(Multiple choices allowed)**	
Loss of resistance to fluid	182 (89)
Loss of resistance to air	35 (17)
Hanging drop method	31 (15)
Ultrasound	3 (2)
Medications used for epidural test dose	
3 mL lidocaine 1.5% + 15 mcg adrenaline	84 (41)
3 mL lidocaine 2%	106 (52)
No test dose used	14 (7)
**Most frequently used medication for postoperative epidural analgesia**	
Opioids	35 (17)
Local anaesthetics	17 (8)
Both opioids and local anaesthetics	150 (74)
Tramadol	2 (1)
**Do you use an epidural catheter for postoperative analgesia?**	
Yes	181 (89)
-	**n (%)**
No	23 (11)
**Do you use adjuvant medications for spinal block for postoperative analgesia?**	
Yes	80 (40)
No	124 (60)
Medications used	
Fentanyl	57 (70)
Morphine	23 (30)

RA, regional anaesthesia; G, gauge

**Table 3. Ranking of RA Complications from Most to Least Common:** 

**Complication**	**1^st^ rank** **n (%)**	**2^nd^ rank** **n (%)**	**3^rd^ rank** **n (%)**	**4^th^ rank** **n (%)**	**5^th^ rank** **n (%)**	**6^th^ rank** **n (%)**
Local anaesthetic systemic toxicity	0 (0.0%)	0 (0.0%)	2 (1%)	15 (13%)	23 (48%)	15 (50%)
Postspinal headache	137 (66%)	49 (23%)	16 (9%)	3 (2%)	0 (0.0%)	0 (0.0%)
Hematoma (subcutaneous)	36 (18%)	63 (31%)	49 (27%)	20 (17%)	0 (0.0%)	0 (0.0%)
Hemopneumothorax	0 (0.0%)	0 (0.0%)	3 (2%)	1 (1%)	18 (38%)	13 (44%)
Nerve damage	2 (1%)	26 (13%)	56 (31%)	47 (40%)	2 (4%)	1 (3%)
Horner’s syndrome	29 (14%)	65 (32%)	56 (31%)	33 (28%)	5 (10%)	1 (3%)

RA, regional anaesthesia

**Table 4. Postoperative Regional Anaesthesia Methods and Age Limits for Regional Analgesia in Paediatric Patients:** 

-	**n (%)**
**Postoperative RA method in paediatric patients (multiple choices allowed)**	
Spinal	5 (2)
Epidural catheter	7 (3)
Caudal	81 (40)
Peripheral block	83 (41)
I do not prefer regional techniques in children	68 (33)
**Age limit for applying regional analgesia in paediatric patients**	
I do not apply it to very young children	93 (46)
I have no age limit, I apply it to all children	45 (22)
I do not apply regional analgesia to paediatric patients	66 (32)

RA, regional anaesthesia

**Table 5. Examination of the Relationship Between the Institution and Variables:** 

- -		**The institution you work for**				**-** ***P* value** **-**
		**University hospitals**	**Teaching and research hospitals**	**State hospitals**	**Private hospitals**	
		**n (%)**	**n (%)**	**n (%)**	**n (%)**	
Percentage of surgeries performed under regional anaesthesia	20% or less	4 (5.0)	3 (3.8)	3 (7.9)	0 (0.0)	0.026*
	20-40%	8 (10.0)	6 (7.7)	4 (10.5)	3 (37.5	
	40-60%	35 (43.8)	23 (29.5)	17 (44.7)	5 62.5	
	More than 60%	33 (41.3)	46 (59.0)	14 (36.8)	0 0.0	
Postoperative analgesia preference (multiple choices)	Parasetamol and NSAIDs	77 (96.3)	73 (93.6)	35 (92.1)	8 (100.0)	0.681
	IM or IV opioids	36 (45.0)	38 (48.7)	18 (47.4)	6 (75.0)	0.449
	PCA	21 (26.3)	32 (41.0)	13 (34.2)	2 (25.0)	0.246
	Peripheral nerve block or catheter infusion	43 (53.8)	60 (76.9)	23 (60.5)	7 (87.5)	0.009*
	Infiltration analgesia	12 (15.0)	10 (12.8)	1 (2.6)	0 (0.0)	0.161
First choice of analgesia in open abdominal surgery	Epidural	21 (26.3)	43 (55.1)	10 (26.3)	5 (62.5)	0.004* -
	TAP + RSB	51 (63.8)	30 (38.5)	21 (55.3)	3 (37.5)	
	QLB	1 (1.3)	0 (0.0)	3 (7.9)	0 (0.0)	
	ESP	1 (1.3)	2 (2.6)	0 (0.0)	0 (0.0)	
	Other	6 (7.5)	3 (3.8)	4 10.5	0 (0.0)	
Most commonly used technique in peripheral nerve block (PNB)	Blind technique	1 (1.3)	0 (0.0)	1 (2.6)	0 (0.0)	0.000*
	NS	4 (5.0)	7 (9.0)	4 (10.5)	1 (12.5)	
	USG	10 (12.5)	47 (60.3)	16 (42.1)	4 (50.0)	
	USG + NS	63 (78.8)	24 (30.8)	16 (42.1)	3 (375)	
	USG + NS + PM	2 (2.5)	0 (0.0)	1 (2.6)	0 (0.0)	

PNB, peripheral nerve block; NSAIDs, non-steroidal anti-inflammatory drugs; IM, intramuscular; IV, intravenous; PCA, patient-controlled IV or epidural analgesia; TAP, transversus abdominis plane; RSB, rectus sheath block; QLB, quadratus lumborum block; ESP, erector spinae plane; NS, nerve stimulator; USG, ultrasonography; PM, pectoralis major

**Table 6. Regional Anaesthesia Preferences for Upper Extremity, Lower Extremity, Thoracic, and Abdominal Surgery:** 

**Category**	**n (%)**
**Percentage of surgeries performed under regional anaesthesia**	
Less than 20%	10 (5)
20-40%	21 (10)
40-60%	80 (39)
More than 60%	93 (46)
**Postoperative analgesia preferences (multiple choices allowed)**	
Paracetamol and NSAIDs	193 (95)
IM or IV opioids	98 (48)
PCA	68 (33)
Peripheral nerve block or catheter infusion	133 (65)
Infiltration analgesia	23 (11)
**Shoulder surgery analgesic first preference**	
Interscalene block	117 (57)
Supraclavicular + suprascapular block	33 (16)
Interscalene + cervical block	31 (15)
Shoulder infiltration analgesia	3 (2)
Other (paracetamol, tramadol, morphine, and NSAIDs)	20 (10)
**Elbow, forearm, and hand surgery analgesic first preference**	
Interscalene block	6 (3)
Supraclavicular block	26 (13)
Infraclavicular block	69 (34)
Axillary block	100 (50)
**Hip surgery analgesic first preference**	
Spinal, epidural	157 (77)
Lumbar plexus block	4 (2)
Fascia iliaca block	14 (7)
PENG block	28 (14)
**Knee surgery analgesic first preference**	
Spinal, epidural	142 (70)
Fascia iliaca block	2 (1)
Femoral block + IPACK	33 (16)
Adductor canal block + IPACK	25 (12)
LIA	1 (1)
**Thoracic surgery analgesic first preference**	
Thoracic epidural	66 (32)
Thoracic paravertebral	23 (11)
Erector spinae block	69 (34)
PECS and serratus block	20 (10)
Other (paracetamol, tramadol, morphine, and NSAIDs)	26 (13)
**Table 6.Continued**	
**Category**	**n (%)**
**Open abdominal surgery analgesic first preference**	
Epidural	79 (39)
Transversus abdominis and rectus block	105 (52)
Quadratus lumborum block	4 (2)
Erector spinae block	3 (1)
Other (paracetamol, tramadol, morphine, and NSAIDs)	13 (6)

NSAIDs, non-steroidal anti-inflammatory drugs; IM, intramuscular; IV, intravenous; PCA, principal component analysis; IPACK, infiltration between the popliteal artery and capsule of the knee LIA, local infiltration analgesia

**Table 7. Medications and Methods Used for Peripheral Block, Sedation Preferences Before and Intraoperative Period:** 

-	**n (%)**
**Which technique(s) do you most frequently use for PNB (multiple choices allowed)?**	
Blind technique	2 (1)
Neurostimulator	16 (8)
USG	77 (38)
Ultrasound + Neurostimulator	106 (52)
Ultrasound + Neurostimulator + Pressure monitor	3 (1)
**Most frequently used LA agents in PNB?**	
Bupivacaine	5 (3)
Bupivacaine + Lidocaine	92 (45)
Bupivacaine + Prilocaine	103 (50)
Lidocaine	4 (2)
**Most frequently used adjuvants in PNB for extremity surgery?**	
Dexamethasone	56 (37)
Dexmedetomidine	18 (12)
Clonidine	1 (1)
Opioids	62 (41)
Other	14 (9)
**Is intralipid solution available in the operating room?**	
Yes	187 (92)
No	17 (8)
**Do you apply sedation before performing RA?**	
I always apply sedation	151(74)
No, I do not apply sedation	15 (7)
I apply it to selected patients	38 (19)
**Do you apply intraoperative sedation to patients undergoing RA**?	
Yes	153 (75)
No	5 (2)
I apply it to selected patients	46 (23)
**Do you use a peripheral block catheter for postoperative analgesia?**	
Yes, frequently	2 (1)
Yes, occasionally	26 (13)
Yes, rarely	62 (30)
No	114 (56)

PNB, peripheral nerve block; USG, ultrasound; LA, local anaesthetic; RA, regional anaesthesia
